# Supplementary material for: Acceptance and compliance with micronutrient powder (MNP) among children aged 6–23 months in northern Nigeria
Source: PLOS Glob Public Health. 2022 Oct 17;2(10):e0000961. doi: 10.1371/journal.pgph.0000961 (PMC10022258; doi:10.1371/journal.pgph.0000961)
Supplement: S1 Dataset — (ZIP) [file pgph.0000961.s008.zip › S8_Dataset/Spot Checks.zip!/Spot Checks/12072015_A_GU_AS_2_SC.pdf]

SPOT CHECK RECORDING FORM

State: Adamawa LGA: ____Guyuk___ Community: _Purokayo

Name of caregiver (female): Asamma Joseph

Name or caregiver (male): ………………………

Sex of index child: _Male___ Age of index child (in months): _ 19 months__________

Spot Check Information

1. **Date of MNP distribution to household** (circle option below based on LGA name above)**:**

   - **Kebbi:** BirninKebbi&Jega LGA: **June 16**
   - **Kebbi:** Danko-wasagu: **June 17**
   - **Adamawa:**Song, Ganye: **June 18**
   - **Adamawa:**Guyuk **June 19**

2. **Date of Spot Check Observation: ____12/7/2015_____**

3. **Number of sachets remaining**(Ask the caregiver to show you and count the specific number of sachets remaining)**:**

   47

4. **Number of sachets that should remainusing one MNP sachet per day** (Analyst only)**:**

   38

5. **Number of sachets remaining / number of sachets should be remaining**(Analyst only)**:**

_____47___ / ___38______ = __123.7__ %

Probes

Question 1. You have **XX** MNP sachets remaining. Explain how easy or difficult it was to use the MNP product on a daily basis for your child 6 – 23 months? (Circle one answer)

Very Difficult **Difficult** **Easy** **Very Easy**

Question 2. Describe any specific challenges that you faced that made the MNP difficult to use on a daily basis?

After using this product for some days the child refused to take kunu mixed with the product. I was confused and I started mixing the product into solid foods like tuwo (guinea corn meal) and okro soup. After you came today and told me to try it once again I mix the product into kunu and add little sugar, the child eat all the kunu. I now understand how to device means to feed him daily with the product

Question 3. Describe specific aspects of the MNP product that you or your child enjoyed that made it easier to use on a daily basis?
The aspect that makes my job easier is the fact that I am to pick a sachet daily and use for the child. Everyday when I pick one sachet to use and mix it into either kunu or tuwo it makes it easy for me.

Question 4. What questions do you have about the MNP based on your experience using it?

- I do not have questions but I now understand that I suppose to think of different ways of preparing kunu so that my child can accept the product.

Question 5. What specific recommendations do you have that we can do as a program to make the MNP easier to use on a daily basis?

The product is very good even though I skip some days to administer it to my child, I have seen the general improvement in my child diet and all I can say is thank you to federal ministry of health for introducing this product to us.
